# The origin of the phrase comparative psychology: an historical overview

**DOI:** 10.3389/fpsyg.2023.1174115

**Published:** 2023-05-15

**Authors:** Raffaele d’Isa, Charles I. Abramson

**Affiliations:** ^1^Institute of Experimental Neurology (INSPE), Division of Neuroscience (DNS), IRCCS San Raffaele Scientific Institute, Milan, Italy; ^2^Department of Psychology, Oklahoma State University, Stillwater, OK, United States

**Keywords:** comparative psychology, animal psychology, history of science, history of psychology, psychology, origin of word psychology, origin of phrase comparative psychology

## Abstract

Comparative psychology, in its narrow meaning, refers to the study of the similarities and differences in the psychology and behavior of different species. In a broader meaning, it includes comparisons between different biological and socio-cultural groups, such as species, sexes, developmental stages, ages, and ethnicities. This broader meaning originated by extension from the former narrow meaning, which historically was the original meaning of the phrase (interspecies psychological and behavioral comparisons) and which still today is the focus of the field. Currently, comparative psychology is a subject of study in hundreds of universities all over the world. Nevertheless, a question that is often asked but seldom answered is: when did the phrase comparative psychology first appear and where did it come from? In the present work, we tracked down the origins of the phrase comparative psychology. In order to do so, at first we described the origin of the word psychology, coined in the decade 1510–1520 in the Republic of Venice by Dalmatian Renaissance humanist Marko Marulić Splićanin (1450–1524). Then, to explain where, within the phrase comparative psychology, the term comparative came from, we outlined the origin of the use of the word comparative in reference to interspecies comparisons. Finally, we reported the origin of the combination of the words comparative and psychology to form the phrase comparative psychology, the first usage of which was in 1778 by German scholar Michael Hissmann (1752–1784) from the University of Göttingen. Origins of the phrase in Latin, German, French and English languages are described.

## Introduction

Comparative psychology, in its narrow meaning, refers to the study of the similarities and differences in the psychology and behavior of different species, especially between non-human animals and humans, and between non-human animal species. Subsequently, the phrase also acquired a broader meaning, in which comparative refers more generally to an approach rather than specifically to interspecies comparisons. In this sense, it includes comparisons between species, sexes, developmental stages, ages, strains, races, and ethnicities. This broader meaning originated by extension from the former narrow meaning, which historically was the original meaning of the phrase (interspecies psychological and behavioral comparisons) and which still today is the main focus of the field. Currently, comparative psychology is a subject of study in hundreds of universities all over the world. Nevertheless, a question that is often asked but seldom answered is: when did the phrase comparative psychology first appear and where did it come from?

In the present work, we will track down the origins of the phrase comparative psychology. In order to do so, we will at first describe the origin of the word psychology. Then, in order to explain where, within the phrase comparative psychology, the term comparative came from, we will outline the origin of the use of the word comparative in reference to interspecies comparisons. Finally, we will report the origin of the combination of the words comparative and psychology to form the phrase comparative psychology. The origins of the phrase in Latin, German, French, and English languages will be described. The paper will focus on the origin period of comparative psychology, i.e., the period from the first use of the phrase in 1778 to the academic institutionalization of comparative psychology in 1888, when comparative psychology became an official university course in Europe and in America.

## The origin of the term psychology

The term psychology derives from the Greek *psyche* (soul, mind) and *logia* (study). The term was coined in the decade 1510–1520 in the Republic of Venice by Dalmatian Renaissance humanist Marko Marulić Splićanin (1450–1524), known in Latin as Marcus Marulus Spalatensis. Dalmatia, currently a region of Croatia, was at that time part of the Republic of Venice, an Italian state, with Venice as capital city, which existed from 697 to 1797.^1^ Marulić, who was born and died in the Venetian-Dalmatian city of Spalato (currently Split in Croatia), wrote in Latin and in Croatian, making important contributions to both literatures. Indeed, on the one hand, he is the father of Croatian literature, with his epic poem *Judita* (finished in 1501 and published in 1521) being the first long poem in the Croatian language (Gutsche, 1975). On the other hand, he contributed to Renaissance Latin literature with both poems and essays. In one of his essays, he used for the first time the word psychology, which was also included in the title: *Psichiologia de ratione animae humanae* (Psychology: On the Nature of the Human Soul; Krstic, 1964; Brožek, 1999). Unfortunately, this work did not survive up to the present, but it is cited in the biography of Marulić written by his contemporary Frano Božićević (1469–1542), *Vita Marci Maruli Spalatensis* (Life of Marcus Marulus from Spalato), which can be dated between 1524 (Marulić’s death) and 1542 (Božićević’s death). Božićević dedicates the sixth section of the biography to presenting a list of Marulić’s works. The *Psichiologia de ratione animae humanae* is placed in position 9, just before the epic poem *Davidias*, which is currently dated around 1517 (Jovanović, 2005).

The earliest preserved printed works featuring the term psychology are two books published in 1525, just 1 year after the death of Marulić (Janssen and Hubbard, 2021). Both books used the term in the Latin form *psychologia*. Dutch-born theologian Gerhard Schnell (c. 1470–1552), known in Latin as Gerardus Synellius, Abbot at the Abbey of Marienthal in Lower Saxony (at that time part of the Holy Roman Empire and currently part of Germany), used the term psychology to refer to his theories on the soul (*psychologia synelliana*; Schnell, 1525).^2^ Italian physician and philosopher Pier Nicola Castellani (active in the period 1475–1525), in Latin Petrus Nicolaus Faventinus, professor at University of Ferrara (1502–1503) and at University of Pisa (1519–1521), used the term psychology in a work on the immortality of the soul (Castellani, 1525), that he dedicated to Pope Clement VII, born Giulio de’ Medici (1478–1534). According to Castellani, “the whole science about the soul, which is called Psychology, is said by the Greeks to be in the middle between Physics and Metaphysics” and “one part of the science about the soul is that which is natural, physiology, just as Aristotle openly writes in the end of his book On the Motion of Animals and in the first book of his On the Soul, Commentary 15 and the sixth book of his Metaphysics, Commentary 2. Likewise, one part of Psychology is metaphysics, as concerning that aspect of the soul which is not natural, just as Aristotle gives witness in Book 2 of On the Parts of Animals, Chapter 1, and Book 6 of the Metaphysics, Commentary 2” (Castellani, 1525; English translation from: Janssen and Hubbard, 2021). Interestingly, while Schnell employed the term in a totally metaphysical-theological context, in Castellani’s view psychology is part metaphysical and part physical.

About half a century later, in 1574, the term psychology was used in *Quaestiones logicae et ethicae* (Logical and Ethical Issues) by German philosopher Johann Thomas Freig (1543–1583), known in Latin as Johannes Thomas Freigius (Freig, 1574). Importantly, in this work Freig, professor at University of Freiburg from 1570, proposed a disciplinary division of knowledge and he inserted *psychologia* among the physical disciplines (*physica*), in particular under the physics of material bodies (*corporum*),^3^ together with physiology (*physiologia*), medicine (*medicina*), zoology (*historia animalium*), astronomy (*astrologia*), and meteorology (*meteorologia*) (Lamanna, 2010). One year later, in his *Ciceronianus*, Freig dedicated to psychology a chapter entitled *De psychologia et hominis fabrica* (On Psychology and the Structure of Man; Freig, 1575). In the same work, Freig attached as preface the *Catalogus locurum communium* (Catalog of Commonplaces), an updated classification of disciplines, in which he confirmed psychology under the *physica* section, in which he added botanics (*historia plantarum*) and metal mining/metallurgy (*metallica*).

The oldest preserved published book with psychology in its title is *Psychologia: hoc est, De hominis perfectione, animo et in primus ortu hujus, commentationes ac disputationes quorundam theologorum & philosophorum nostra aetatis* (Psychology: That Is to Say, On the Perfection of Man, On the Rational Mind and Particularly On Its Origin, with the Comments and Discussions of Certain Theologians and Philosophers of Our Age), published in 1590 in Marburg by German scholastic philosopher Rudolf Göckel (1547–1628), known in Latin as Rodolphus Goclenius (Göckel, 1590), who was professor at Marburg University from 1581 (Cellamare, 2015).

In the 17th century the use of the term psychology spread, but it was totally ignored by the major philosophers that wrote of psychological processes, including René Descartes (1596–1650), Thomas Hobbes (1588–1679), John Locke (1632–1704) and Nicolas Malebranche (1638–1715). Nevertheless, the term was reported in several other sources, especially from the medical area.

In 1626, the German encyclopedist Johann Heinrich Alsted (1588–1638), in Latin Johannes Henricus Alstedius, published *Compendium Lexici Philosophici* (Compendium of Philosophical Lexicon), in which he underlined how psychology, as anatomy, is a part of physiology (Alsted, 1626). A few decades later, German philosopher and historian Johannes Lütkeschwager (1597–1658), in Latin Johannes Micraelius, included the word *psychologia* in his dictionary *Lexicon philosophicum* (Philosophical Lexicon), defining it as doctrine of the soul (Lütkeschwager, 1653), while at the end of the century, the Dutch physician and entomologist Steven Blankaart (1650–1704), in Latin Stephanus Blancardus, included *psychologia* (spelled in Greek characters) in the second edition of his medical dictionary, the *Lexicon Novum Medicum Graeco-Latinum* (New Greek-Latin Medical Lexicon; Blankaart, 1690).

The term became more relevant at the beginning of the 18th century. In 1703 John Broughton (1673/1674–1720), chaplain of the Duke of Marlborough (England), published *Psychologia: Or, an Account of the Nature of the Rational Soul*, a book in English in which he reflects on the mind-body problem (Broughton, 1703). This text was important in promoting the word *psychologia* among English readers. Nevertheless, the topics discussed belong more to what known today as philosophy of mind rather than to psychology. In this work, Broughton takes an anti-materialistic position and argues that a material substance (the body) cannot be capable in itself of thought, for which an immaterial substance is required (the soul).

In the 1730s, German philosopher and protopsychologist Christian Wolff (1699–1754), published *Psychologia empirica* (1732) and *Psychologia rationalis* (1734), in which the ideas of, respectively, empirical psychology and rational psychology were presented (Wolff, 1732, 1734). While rational psychology is the investigation of the soul through *a priori* deductive reasoning and logic, empirical psychology is based instead on knowledge derived from actual experience rather than from an *a priori* concept. The first text, for which the extended title is *Psychologia empirica methodo scientifica pertractata* (Empirical Psychology Examined by the Scientific Method), is considered by many the ground zero of modern scientific psychology (Luccio, 2013; Klempe, 2020a; de Freitas Araujo, 2021). Wolff’s work had a great influence during the Enlightenment (Klempe, 2020b) and the French encyclopedist Denis Diderot (1713–1784), reporting Wolff’s subdivision, included in his epochal *Encyclopédie* an article dedicated to the word psychology (psychologie), which widely increased the visibility of the term (Diderot, 1765).

In the first years of the 18th century scholars started to use more often the term psychology, which within a few decades became the most common term to refer to the study of mental processes.

## The origin of the use of the word comparative to refer to comparisons across species

Comparative psychology, in a broader sense, is the study of psychological and behavioral processes through comparisons between species, sexes, developmental stages, ages, strains, races, or ethnicities. In a narrow sense, it is the study of the similarities and differences in psychological and behavioral processes of different species. This narrow sense is also its original meaning. Historically, within the phrase comparative psychology, where did the word comparative come from? The phrase comparative psychology was created, by analogy, on the model of the pre-existing phrase comparative anatomy, which is the study of similarities and differences in the anatomy of different species. Who and when started using the word comparative in relation to comparisons across species?

**Figure 1:**
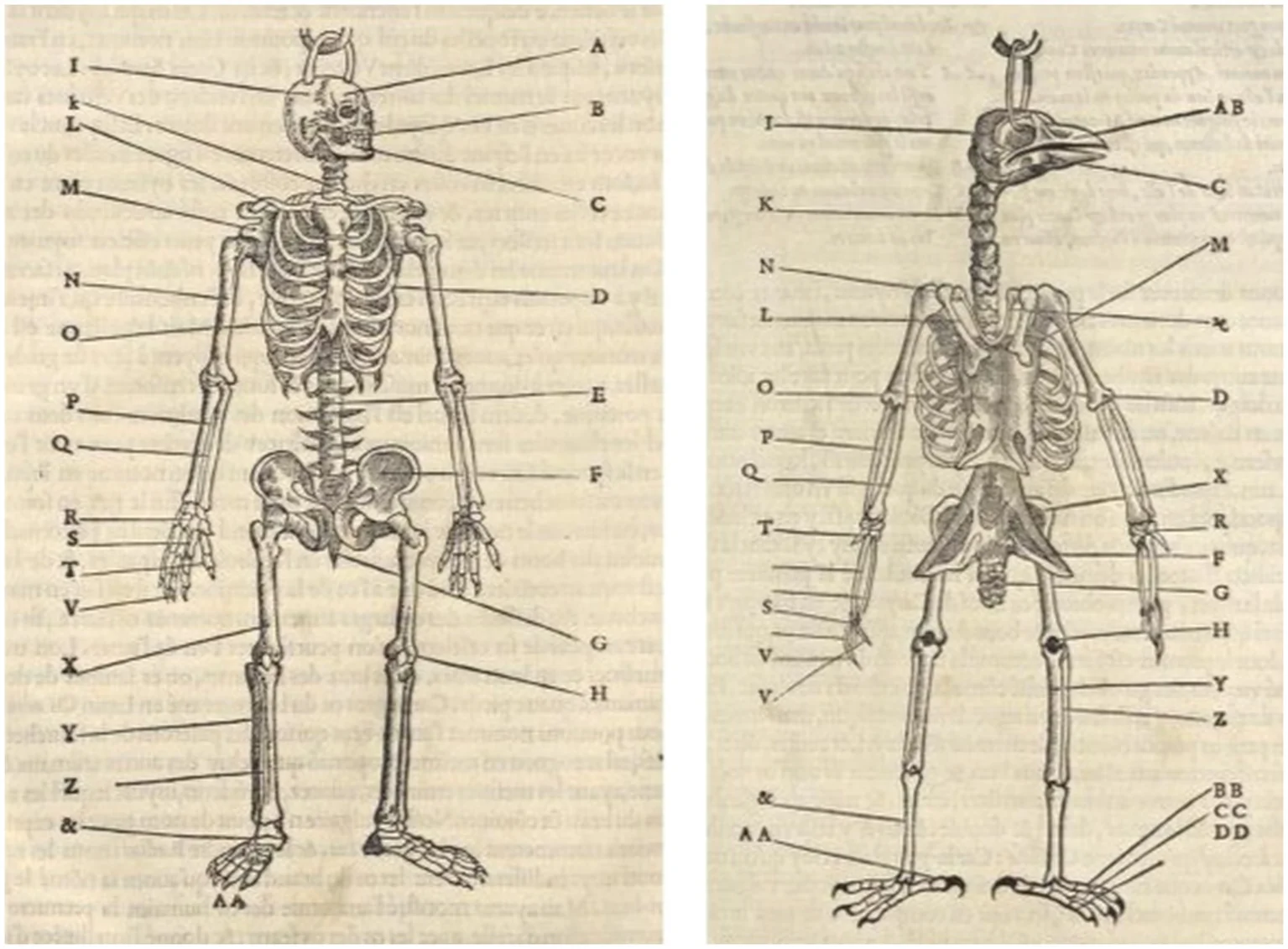
The comparative approach: Pierre Belon’s comparison between the bird skeleton and the human skeleton (1555). The comparative approach (meant as interspecies comparisons) was first brought to a formal level by French naturalist Pierre Belon (1517–1564). In *Histoire de la nature des oyseaux* (Natural History of Birds), Belon made a direct comparison between the bird skeleton (on the right) and the human skeleton (on the left), indicating with the same letter the homologous bones (Belon, 1555).

Modern comparative anatomy started in the 16th century with the work of the French naturalist Pierre Belon (1517–1564), in Latin Petrus Bellonius Cenomanus, who in *De aquatilibus* (On Aquatic Animals) reported the description of over 170 species of fish, aquatic mammals (including whale, dolphin, otter, and hippopotamus) and aquatic reptiles (including crocodile and sea turtle) with illustrations (Belon, 1553), and in *Histoire de la nature des oyseaux* (Natural History of Birds) compared the figures of the bird skeleton and the human skeleton, marking with a same letter the homologous bones (Belon, 1555; Figure 1).

The field of comparative anatomy was further consolidated by the Italian surgeon Marco Aurelio Severino (1580–1656), in Latin Marcus Aurelius Severinus, who wrote *Zootomia democritea* (Democritean Zoological Anatomy; Severino, 1645) and by the English physician Edward Tyson (1651–1708), who wrote *Orang-Outang, sive Homo Sylvestris: or, the Anatomy of a Pygmie Compared with that of a Monkey, an Ape, and a Man* (Tyson, 1699).

The phrase comparative anatomy appeared at the beginning of the 17th century. The English philosopher Francis Bacon (1561–1626) used it in Latin (*anatomia comparata*) in 1623, but with a different meaning, since he described it as the study of the different appearance, condition, and position of anatomical parts in different men (Bacon, 1623; English translation: Bacon, 1674). The first to attribute the current meaning to the phrase comparative anatomy was the English physician Thomas Willis (1621–1675), professor at Oxford University, in *Cerebri anatome* (Anatomy of the Brain), referring to it as the study of the anatomical similarities and differences in various animals, among them and with man (Willis, 1664). Other authors employed the phrase comparative anatomy in the 17th century (for example: Grew, 1672, 1675, 1681; Lister, 1672; Tyson, 1698; Wallis, 1699). However, the phrase became more popular in the first half of the 18th century, being used by German naturalist Michael Bernhard Valentini (1657–1729) in the monumental *Amphitheatrum zootomicum* (Amphitheater of Zoological Anatomy; Valentini, 1720), by Scottish physician Alexander Monro (1697–1767), who became the first professor of anatomy of the University of Edinburgh in 1720, in *An Essay on Comparative Anatomy* (Monro, 1744), and by Swedish naturalist Carl Nilsson Linnaeus (1707–1771), who is considered the father of modern biological taxonomy, the scientific classification of living organisms (Linnaeus, 1748).

## The origin of the phrase comparative psychology

The phrase comparative psychology appeared in the second half of the 18th century. As we mentioned, it was formed on the model of the phrase comparative anatomy, which at that time had already become popular among scholars. This choice reflected the determination to place this type of psychology among the medical sciences, rather than among the metaphysical disciplines. Where and when did the phrase comparative psychology appear for the first time?

According to current knowledge, the first use in a printed publication was in Latin (*psychologia comparata*) by German materialistic philosopher Michael Hissmann (1752–1784), professor at University of Göttingen from 1782, in the bibliographic book *Anleitung zur Kenntniss der auserlesenen Litteratur in allen theilen der Philosophie* (Guide to the Knowledge of Selected Literature in All Parts of Philosophy), published in 1778 in Göttingen and in the small town of Lemgo (Hissmann, 1778). In 1765 British physician John Gregory (1724–1773), professor of Edinburgh University from 1766 and named first physician of Scotland by King George III (1738–1820) in the same year, had published *A Comparative View of the State and Faculty of Man with those of the Animal World* (Gregory, 1765). In this work, Gregory used the word comparative in reference to interspecies psychology, but, instead of using the word psychology, he used the periphrasis “state and faculty.” Three years later, French encyclopedist Charles-Georges Le Roy (1723–1789) published a book on animal behavior, *Lettres sur les animaux* (Letters on Animals; Le Roy, 1768). Importantly, analogously to Gregory, Le Roy also never used in his work the word psychology. It was only German scholar Michael Hissmann that, to describe the subject covered by Gregory’s book and by Le Roy’s book, which had just been translated in German as *Briefe über die Thiere und den Menschen* (Letters on Animals and Men; Le Roy, 1775), employed the phrase *psychologia comparata* (Hissmann, 1778). The phrase was subsequently included also in the second edition of Hissmann’s work (Hissmann, 1790).

At the beginning of the 19th century, the phrase was first used in German (vergleichende psychologie) by physician and physiological anthropologist Wilhelm Liebsch (c. 1778–1808/1820) from Göttingen (Liebsch, 1808). In his *Grundriss der Anthropologie physiologisch und nach einem neuen Plane bearbeitet* (Foundations of Anthropology Treated Physiologically and According to a New Plan), Liebsch employed the phrase in reference to the study of the mental characteristics of non-human animals compared to the ones of humans (Liebsch, 1808).

A few years later, in 1812, the phrase was used another time in Latin, again by a German author, philosopher and psychologist Johann Christoph Hoffbauer (1766–1827), professor at University of Halle from 1794. Hoffbauer’s *Einiges über die Psychologia comparata* (Remarks on Comparative Psychology) is the first essay in history bearing the phrase comparative psychology in the title and explicitly dedicated to the topic (Hoffbauer, 1812). In this essay Hoffbauer refers to comparative psychology as a study of animal psychology that can shed light on human psychology. In his words: “Shouldn’t a psychological observation, which we did not consider unworthy of animals, also open our eyes more to some things about ourselves that we constantly see coming and going by without looking at them seriously? I always believe that the Psychologia comparata, as I want to call it first of all, can be just as beneficial for the natural science of the human soul as observations on animal bodies have become for the natural science of the human body” (Hoffbauer, 1812).^4^ The essay was published in *Beyträge zur Beförderung einer Kurmethode auf psychischem Wege* (Contributions to Promoting a Method of Treatment Using Psychic Approaches), a journal that Hoffbauer founded in 1808 together with German neuroanatomist and psychiatrist Johann Christian Reil (1759–1813), professor of medicine at University of Halle from 1787, official physician of the city of Halle (Stadtphysikus) and first to provide a detailed description of the insular lobe of the brain, which today is also named Reil’s insula (*insula Reili*) in his honor (Marneros, 2008). Reil is also one of the first authors to utilize the word psychiatry (in German: psychiaterie),^5^ in an essay published in the same journal co-edited with Hoffbauer (Reil, 1808), in which he argued that psychiatry should be established as a new medical specialty and that medicine should be divided into three main branches: surgery, pharmacy (internal medicine) and psychiatry (Marneros, 2008).

In 1816 German anatomist and physiologist Friedrich Tiedemann (1781–1861), professor at University of Heidelberg, in his *Anatomie und Bildungsgeschichte des Gehirns im Fetus des Menschen: nebst einer vergleichenden Darstellung des Hirnbaues in den Thieren* (Anatomy and Developmental History of the Brain in the Human Fetus: Together with a Comparative Presentation of the Brain Structure in Animals) used the phrase in German but in a slightly different form than the one used by Liebsch (vergleichenden psychologie), writing: “Just as we must achieve through the examination of the nervous system and the brain of the animals the knowledge of the gradual formation and composition of the brain, so we also need a comparative psychology to recognize the role and action of the parts of the brain” (Tiedemann, 1816).^4^

This alternative German version of the phrase was then employed by Austrian veterinarian Georg Franz Eckel (1797–1869), from the Veterinary Medicine Institute of Vienna (of which he would subsequently become Director), in his book on veterinary medicine (Eckel, 1823).

Between the 1820s and the 1840s German psychiatrists Heinrich Philipp August Damerow (1798–1866), Carl Friedrich Flemming (1799–1880), Ludwig Buzorini (1801–1854), and Friedrich Wilhelm Hagen (1814–1888) all used the phrase, returning to adopt Liebsch’s version (vergleichende psychologie), which thereon became consolidated in the German language (Damerow, 1829; Flemming, 1830; Buzorini, 1832; Hagen, 1841).

In Austria, after Eckel, Austrian zoologist Ludwig Karl Schmarda (1819–1908) also preferred Tiedemann’s version of the phrase, choosing it for his *Der thierische Trieb vom naturhistorischen Standpuncte betrachtet* (The Animal Drive Examined from the Natural History Point of View; Schmarda, 1843). Liebsch’s version of the phrase started to be used only subsequently, by Austrian psychiatrist Ernst Freiherrn von Feuchtersleben (1806–1849), who employed it, along with Tiedemann’s version, in his influential *Lehrbuch der ärztlichen Seelenheilkunde* (Textbook of Medical Psychiatry; von Feuchtersleben, 1845).

The phrase comparative psychology was first used in Italian (psicologia comparata) in 1827 by philosopher and historian of philosophy Baldassarre Poli (1795–1883). In 1826 Poli, driven by interest in biological psychology, wrote two articles on craniology (also known as phrenology), the discipline, founded by German physician Franz Joseph Gall (1758–1828), which tried to establish connections between mental faculties and the shape of the cranial bones (Poli, 1826a,b), expressing several doubts on the principles exposed by Gall (Poli, 1826b). The following year, Poli published the book *Saggio filosofico sopra la scuola de’ moderni filosofi-naturalisti coll’analisi dell’organologia, della craniologia, della fisiognomonia, della psicologia comparata e con una teorica delle idee e dei sentimenti* (Philosophical Essay on the School of the Modern Natural Philosophers with the Analysis of Organology, of Craniology, of Physiognomy, of Comparative Psychology and with a Theory of Ideas and Feelings), which is the first book ever with the phrase comparative psychology in its title. Within this book, in the chapter *Della scienza della psicologia comparata. Origine, principj, critica, verità e utile applicazione della psicologia comparata* (Of the science of comparative psychology. Origin, principles, critique, truthfulness and useful application of comparative psychology), Poli defines comparative psychology as “the science that studies and analyzes the instincts, the functions and the habits of beasts in relation to the analogous human faculties, with the aim to explain better the phenomena of thought and feeling in man” (Poli, 1827).^4^ Importantly, Poli is also the first author to utilize extensively the phrase comparative psychology. While Hissmann, Liebsch, Tiedemann and Eckel used it only once in their books (Hissmann, 1778; Liebsch, 1808; Tiedemann, 1816; Eckel, 1823) and Hoffbauer twice in his essay (Hoffbauer, 1812), Poli used the phrase 30 times in his book (Poli, 1827).

Some decades later, Poli, who had become professor at the University of Padova and President of the Veneto Institute of Science, Letters and Arts,^6^ further contributed to comparative psychology by publishing an essay on the relationship between cerebral convolutions and cognitive abilities (Poli, 1855), a critical review based on the dissections of the French neuroanatomist François Leuret (1797–1851), who compared the nervous system of a wide variety of species, including invertebrates, fish, reptiles, birds, and mammals (Leuret, 1839). Interestingly, Poli’s publications of the 1820s set the ground for the birth of a modern (organized, experience-based and biologically rooted) general psychology, which in Italy started with the work of experimental physicist Francesco Zantedeschi (1797–1873). In 1832 Zantedeschi published *Elementi di psicologia empirica* (Elements of Empirical Psychology; Zantedeschi, 1832), followed 3 years later by a second edition entitled simply *Elementi di psicologia* (Elements of Psychology; Zantedeschi, 1835).

In 1877, anthropologist and comparative psychologist Tito Vignoli (1824–1914), pupil of zoologist Paolo Savi (1798–1871) and professor at the Royal Academy of Science and Letters of Milan^7^ from 1874 (Canadelli, 2010, 2013, 2015), published *Della legge fondamentale dell’intelligenza nel regno animale: saggio di psicologia comparata* (On the Fundamental Law of Intelligence in the Animal Kingdom: Essay of Comparative Psychology), the first Italian book entirely and explicitly dedicated to comparative psychology (Vignoli, 1877). Interestingly, Vignoli subsequently became director of the Museum of Natural History of Milan, first comparative psychologist to hold this role, and maintained this position from 1893 until his retirement in 1911 (Canadelli, 2010).

The phrase comparative psychology was first used in French (psychologie comparée) in 1808 by physician Jean-Baptiste Nacquart (1780–1854), who, in his *Traité sur la nouvelle physiologie du cerveau* (Treatise on the New Physiology of the Brain), referring to knowledge on animal intelligence, defines it “a branch of comparative psychology” (Nacquart, 1808).^4^ In the same book, Nacquart employs the term a second time, stating that “comparative psychology must clarify very much the history of our faculties.”

In 1823 Tiedemann’s book on the fetal development of the brain was translated in French by the physician Antoine Jacques Louis Jourdan (1788–1848), leading to a second appearance of the phrase psychologie comparée, although this time it was not an original use by a French author, but rather a translated phrase in the French edition of a German text (Tiedemann, 1823).

In 1825, the French phrase appeared in a work by Gall, but only when citing literally the comment of Tiedemann on comparative psychology (Gall, 1825). This demonstrates that Gall knew the phrase, although in the rest of the book he never used it. Also in this case, as for Jourdan, the phrase was just a pure translation of the quote from Tiedemann’s work from 1816.

In 1831, French physician Joseph Vimont (1795–1857), from the Sorbonne University of Paris, started publishing his monumental *Traité de phrénologie humaine et comparée* (Treatise on Human and Comparative Phrenology), which included four volumes published over the course of 1831–1835 (Atlas Volume: Vimont, 1831; Text Volume 1: Vimont, 1832; Text Volume 2: Vimont, 1833; Text Volume 3: Vimont, 1835). Although phrenology comes from the Greek *phren* (mind), and is therefore, etymologically, another word for psychology, as defined by Gall, the founder of the discipline, phrenology refers more specifically to the study of the skull than of the brain, and Vimont’s work is more anatomical than psychological. Indeed, Vimont’s work represents the most complete and detailed comparative cranial anatomy ever published up to that time. However, the phrase comparative psychology was never used in his volumes.

Actually, after the occasional usage by Nacquart in 1808, it took a relatively long time, up to the mid-1830s, to see the phrase used again by an original French author. In 1836, the phrase was employed by physician Louis François Lélut (1804–1877), professor at Sorbonne University of Paris (Lélut, 1836). Outlining the fields of observation of psychology, Lélut mentions four types of comparative psychology: psychologie comparée des espèces animales (comparative psychology of animal species), psychologie comparée des races humaines (comparative psychology of human races), psychologie comparée des âges de l’homme (comparative psychology of human ages), and psychologie comparée des maladies (comparative psychology of mental pathologies), which he also defines psychologie pathologique (pathological psychology).

Similarly, in 1846 French physician Pierre-Nicolas Gerdy (1797–1856) mentions “comparative psychology of ages, of sexes, of people, of different social conditions, of different levels of civilization and of animals” (Gerdy, 1846).^4^

In 1864 neurophysiologist Pierre Flourens (1794–1867), who, under the supervision of naturalist George Cuvier (1769–1832), had performed a series of pioneering experiments on the brain functions of pigeons (Flourens, 1825) and who had become professor at Muséum National d’Histoire Naturelle in 1832 and at Collège de France in 1855, published the first book ever with the sole phrase comparative psychology as its title: *Psychologie comparée* (Comparative Psychology; Flourens, 1864).

A few years after Flourens’s works, philosopher Henri Joly (1839–1925) published two books entirely dedicated to comparative psychology: *L’Instinct: ses rapports avec la vie et avec l’intelligence. Essai de psychologie comparée* (Instinct: its Relantionships with Life and Intelligence. Essay of Comparative Psychology; Joly, 1869) and *Psychologie comparée. L’homme et l’animal* (Comparative Psychology. Man and Animal; Joly, 1877). In the same year of the publication of Joly’s second book, doctorate student Alfred Victor Espinas (1844–1922) chose comparative psychology as topic of his final thesis, which he discussed at Sorbonne University of Paris in June 1877 and published in form of book in October 1877: *Des sociétés animales: étude de psychologie comparée* (Of Animal Societies: Essay of Comparative Psychology; Espinas, 1877). Interestingly, from 1881 to 1883 Joly taught comparative psychology, within the course of philosophy, at Sorbonne University of Paris, before becoming in 1887 professor of criminal science in the same university and continuing his academic career as a criminologist.

In February 1888 psychologist Théodule-Armand Ribot (1839–1916) became Professor of Experimental and Comparative Psychology at the Collège de France in Paris, a chair that had just been created in December 1887 and that Ribot won with 20 votes to 8 against Joly, continuously holding this position until 1901 when he retired (Nicolas and Murray, 1999, 2000; Nicolas, 2000; Nicolas and Charvillat, 2001). Just 1 year after obtaining this chair, Ribot also contributed to the foundation, at Sorbonne University, of the first French experimental human psychology laboratory (named Laboratory of Physiological Psychology), the direction of which was assigned to physiologist and psychologist Henry-Étienne Beaunis (1830–1921; Nicolas and Murray, 1999).

In the English language, the phrase comparative psychology was first used in 1821 by British entomologist William Sharp Macleay (1792–1865) from Trinity College in Cambridge, in the first volume of his capital work *Horae Entomologicae; or, Essays on the Annulose Animals* (Macleay, 1821). In 1826 the phrase appeared in the English edition of Tiedemann’s aforementioned book on fetal neurodevelopment, which was translated in English by William Bennett, from the 1823 French translation of Jourdan (Tiedemann, 1826). In the following decade, British physiologist Peter Mark Roget (1779–1869), from 1834 professor at the Royal Institution of Great Britain in London, employed the phrase in his treatise on physiology and phrenology, which was published in a British edition in Edinburgh (Roget, 1838) and in an American edition in Philadelphia (Roget, 1839).

Soon after, the phrase was used by British psychiatrist and ethnologist James Cowles Prichard (1786–1848), in reference to comparisons between different human races and ethnicities, in his influential *Researches Into the Physical History of Mankind* (Prichard, 1841).^8^ British physician Daniel Noble (1810–1885), from the Royal College of Surgeons of England, used the phrase, in his book *The Brain and its Physiology*, to describe the findings of Gall: “to whom we are under great obligations for the advances which he made in comparative psychology, and in the comparative physiology of the nervous system” (Noble, 1846). British physician Robert Dunn (1799–1877) employed the phrase in an article on physiological psychology (Dunn, 1856), reprinted 2 years later in his book *Essay on Psychological Physiology* (Dunn, 1858).

In the mentioned works by Macleay, Roget, Prichard, Noble, and Dunn, the phrase comparative psychology was used only once and almost incidentally. It was only at the end of the 1850s that German zoologist David Friedrich Weinland (1829–1915), at that time researcher at Harvard University in Cambridge (Massachussetts, United States), wrote the first English essay explicitly dedicated to comparative psychology, with the phrase comparative animal psychology employed in the title (Weinland, 1858). Two years later, in his pioneering book on bees, British entomologist James Samuelson (1829–1918) wrote an introduction to comparative psychology, underlining the “necessity for the study of comparative psychology, or the science of mind in animals” (Samuelson, 1860).

The first book in English with the phrase comparative psychology in its title was *Chapters on Man. With the Outlines of a Science of Comparative Psychology*, published in 1868 by British anthropologist Charles Staniland Wake (1835–1910), who in 1871 became the first Director of the Anthropological Institute of Great Britain and Ireland (Wake, 1868). In 1869, British psychologist and anthropologist John William Jackson (1809–1871), in his *Inaugural Address to the Psychological Association of Glasgow*, eloquently exposed the aims of comparative psychology: “comparative psychology must embrace the entire animate scale, with all its diversified classes, orders, genera, and species of sentient being. What is a brute? How does he differ from a man? By what process of subtraction shall we define his lower place in the great scheme of conscious existence? Are his specialities reflected in his organization? From the worm to the lion, is brute mind emblemed in brute structure; and if so, shall we ever prevail to read it of with precision? Are the teeth and talons of the tiger simply its ferocity and cruelty, ultimated in predatory instrumentalities? Is the dove a fair embodiment of love and gentleness? and are opposite qualities equally reflected in the structure of the eagle and the falcon? This, again, brings us back to the connexion between mental aptitudes and organic conditions” (Jackson, 1869).

In the 1870s, the phrase comparative psychology was used several times by British physician and botanist William Lauder Lindsay (1829–1880), from the Murray Royal Institution in Perth (Scotland, United Kingdom), who published several articles (Lindsay, 1871a,b,c, 1872, 1874, 1876, 1877), and subsequently a book (Lindsay, 1879), entirely dedicated to comparative psychology. Most of these articles contain the explicit phrase (Lindsay, 1871a,b, 1874, 1877), and in the first article Lindsay provides a definition for comparative psychology: “the Science of Mind in all classes of Animals, including Man, and in the lower animals specially as contrasted with Man” (Lindsay, 1871a). Importantly, in the book, the monumental *Mind in the Lower Animals in Health and Disease* (a work consisting of two volumes for a total of more than 1,150 pages, based on the behavior of 908 species belonging to 516 genera), Lindsay employs the phrase 37 times and the first chapter is entitled *Comparative Psychology, General Considerations, Including the Methods of Inquiry* (Lindsay, 1879). Lindsay was the most important British comparative psychologist of the 1870s and, after his premature death in 1880, his groundbreaking work had a strong influence on the following generation of comparative psychologists.

In the same year of the publication of Lindsay’s book, British evolutionary biologist George John Romanes (1848–1894), friend of the father of the evolution theory Charles Darwin (1809–1882), positively reviewed Lindsay’s book in *Nature*: “In so extensive a work by so well-known a man there is, as we should expect, a great deal that is both of interest and value” (Romanes, 1879). In the 1880s, Romanes published his own books on comparative psychology, *Animal Intelligence* (Romanes, 1882) and *Mental Evolution in Animals* (Romanes, 1883), two milestone texts that became extremely popular and that both obtained multiple editions over the course of the decade.

In the 1880s, the subject gave rise to a rapidly growing interest and the phrase comparative psychology started to appear in the official names of institutions and university titles. In 1885, the first scientific society dedicated to comparative psychology was founded in Montreal (Quebec, Canada), the Association for the Study of Comparative Psychology, on proposal of the Canadian physiologist and psychologist Thomas Wesley Mills (1847–1915) from McGill University (Mills, 1888, 1897; Murray, 1990). In 1886 Italian psychologist Giuseppe Sergi (1841–1936), who had just founded in 1885 the first human psychology experimental laboratory in Italy (Misiak and Staudt, 1954),^9^ asked for the institution of a course of Experimental and Comparative Psychology at the University of Rome, proposing a program for the course (Sergi, 1886; reprinted in: Sergi, 1987). In 1887, the Collège de France in Paris created the professorship of Experimental and Comparative Psychology, which in February 1888 was assigned to psychologist Théodule-Armand Ribot (Ribot, 1888; Nicolas and Murray, 2000), the first comparative psychology professor in Europe and in the world. In June 1888, the American psychologist Joseph Jastrow (1863–1944) became Professor of Experimental and Comparative Psychology at University of Wisconsin in Madison (Wisconsin, United States), a chair that was established especially for him (Cadwallader, 1987), which made him the first comparative psychology professor in America. As declared by Jastrow in his autobiography, *Animal Intelligence* by George Romanes was adopted as the first textbook for his course (Jastrow, 1930).

In America, the rise of comparative psychology was particularly fast. In 1878, psychologist John Bascom (1827–1911) was the first American author to publish a book explicitly dedicated to comparative psychology (Johnston, 2003), with the title containing the phrase: *Comparative Psychology; Or, The Growth and Grades of Intelligence* (Bascom, 1878). In this work, Bascom proposed the idea of a gradient of consciousness and of cognitive ability across animal species: “By comparative psychology we understand a knowledge of intelligence, of conscious activity, as it exists in all accessible forms of lives, a tracing of its development in its several stages through the entire animal kingdom. This is our present effort, a discussion of the growth and grades of intelligence in the world about us” (Bascom, 1878). Just 10 years after the first American book on comparative psychology, the first American professorship of comparative psychology was officially established (Jastrow, 1888).

Indeed, the rapid increase of popularity and the academic institutionalization in the 1880s laid the ground for the flourishing of the golden age of comparative psychology in the following decades. Table 1 presents a list of selected publications featuring the phrase comparative psychology from the period described in the present paragraph (1778–1888).

**Table 1:** Selected publications featuring the phrase comparative psychology in the period 1778–1888.

Author	Citation
Michael Hissmann1752–1784	Hissmann (1778)
Wilhelm Liebschc. 1778–1808/1820	Liebsch (1808)
Jean-Baptiste Nacquart1780–1854	Nacquart (1808)
Johann Christoph Hoffbauer1766–1827	Hoffbauer (1812)
Friedrich Tiedemann1781–1861	Tiedemann (1816)
William Sharp Macleay1792–1865	Macleay (1821)
Franz Eckel1797–1869	Eckel (1823)
Baldassarre Poli1795–1883	Poli (1827)
Heinrich Philipp August Damerow1798–1866	Damerow (1829)
Carl Friedrich Flemming1799–1880	Flemming (1830)
Ludwig Buzorini1801–1854	Buzorini (1832)
Louis François Lélut1804–1877	Lélut (1836)
Peter Mark Roget1779–1869	Roget (1838, 1839)
James Cowles Prichard1786–1848	Prichard (1841)
Wilhelm Hagen1814–1888	Hagen (1841)
Karl Friedrich Burdach1776–1847	Burdach (1842a,b)
Ludwig Karl Schmarda1819–1908	Schmarda (1843)
Ernst Freiherrn von Feuchtersleben1806–1849	von Feuchtersleben (1845)
Daniel Noble1810–1885	Noble (1846)
Pierre-Nicolas Gerdy1797–1856	Gerdy (1846)
Robert Dunn1799–1877	Dunn (1856, 1858)
David Friedrich Weinland1829–1915	Weinland (1858)
James Samuelson1829–1918	Samuelson (1860)
Pierre Flourens1794–1867	Flourens (1861, 1864)
Charles Staniland Wake1835–1910	Wake (1868)
John William Jackson1809–1871	Jackson (1869)
Henri Joly1839–1925	Joly (1869, 1877)
William Lauder Lindsay1829–1880	Lindsay (1871a,b,c, 1872, 1874, 1877, 1879)
Tito Vignoli1824–1914	Vignoli (1877)
Alfred Victor Espinas1844–1922	Espinas (1877)
John Bascom1827–1911	Bascom (1878)
George John Romanes1848–1894	Romanes (1878, 1879, 1882, 1883, 1884)
Grant Allen1848–1899	Allen (1879)
Giuseppe Sergi1841–1936	Sergi (1881, 1886)
Thomas Wesley Mills1847–1915	Mills (1887, 1888)
Théodule Ribot1839–1916	Ribot (1888)
Joseph Jastrow1863–1944	Jastrow (1888)

The table presents a list of selected publications featuring the phrase comparative psychology from the origin period of comparative psychology (i.e., the period from the first use of the phrase in 1778 to the academic institutionalization of comparative psychology in 1888, when comparative psychology became an official university course in Europe and in America).

## Concluding remarks

For the present paper, we performed extensive research through several digital databases, including WorldCat, the Wellcome Collection, the Biodiversity Heritage Library, the HathiTrust Digital Library, the National Library of Scotland, Bibliothèques d’Université Paris Cité, PennLibraries, Deutsches Textarchiv, Bayerische Staatsbibliothek, Numistral, PubMed, APA PsychInfo, APA PsychArticles, and Google Scholar. At present, practically all the main texts have been digitized. These tools allowed a depth of search for the current history paper that would never have been possible just a couple of decades ago. Nevertheless, it is important to underline that the present work is based on current knowledge. The oldest printed usages for the phrase comparative psychology are contained in a book published in Germany in 1778 (Hissmann, 1778), whose second edition was published 12 years later (Hissmann, 1790), and in two books from 1808, one published in Germany (Liebsch, 1808) and one in France (Nacquart, 1808). Notably, Hissmann, Liebsch, and Nacquart use the expression as if it was already existing and they do not present themselves as the authors of the phrase. It is hence our opinion that the phrase comparative psychology was already used, at least orally, previously, in the period 1760–1777 or maybe even before. Although it is currently very extensive, the digitization of historical texts is an on-going process. It is possible that in future new books will be discovered and/or made digitally available. In this case, it could be possible to update and predate the current known usages for the phrase comparative psychology.

## Author contributions

RdI and CIA conceived the idea of the manuscript. RdI wrote the first draft of the manuscript. RdI and CIA discussed, revised and added to the manuscript. Both authors approved the final submitted version.

## Funding

CIA’s participation to the present work was funded in part by the National Science Foundation (NSF) of the United States. Grants: NSF Research Experiences for Undergraduates (REU) grant 1950805 and NSF Partnerships for International Research and Education (PIRE) grant 1545803.

## Conflict of interest

The authors declare that the research was conducted in the absence of any commercial or financial relationships that could be construed as a potential conflict of interest.

## Correction note

This article has been corrected with minor changes (one reference was corrected). These changes do not impact the scientific content of the article.

## Publisher’s note

All claims expressed in this article are solely those of the authors and do not necessarily represent those of their affiliated organizations, or those of the publisher, the editors and the reviewers. Any product that may be evaluated in this article, or claim that may be made by its manufacturer, is not guaranteed or endorsed by the publisher.

## Footnotes

^1^In particular, Venetian Dalmatia (in Latin *Dalmatia Veneta*) existed from 1409 to 1797 as a province of the Republic of Venice.

^2^A coeval of Schnell, chronicler Eggerik Beninga (1490–1562), reports that Schnell’s work was completed in 1512 (Beninga, 1723). In this case, the work could be earlier than Marulić’s. If Beninga’s claim is to be considered true, it could be that some years elapsed between completion and publication of Schnell’s work or it could be that the 1525 edition is a reprint. In either case, we cannot know if the word *psychologia* was present in the 1512 version. We can only know for sure that it is present in the 1525 book.

^3^Freig subdivided physics (*physica*) into two parts (Lamanna, 2010): physics of senses (*sensum*), which included for example optics (*optica*), and physics of material bodies (*corporum*). Interestingly, psychology was included in the latter category.

^4^English translation by Raffaele d’Isa.

^5^The word psychiatry had been coined just few years earlier, in 1800, in German (psychiatrie), by physiologist Karl Friedrich Burdach (1776–1847), who, in the same work, also coined the word biology (in German: biologie) (Burdach, 1800). Interestingly, after having become professor at several important Northern Europe universities, namely University of Leipzig in 1807, University of Dorpat (now Tartu in Estonia) in 1811 and University of Königsberg (now Kaliningrad in Russia) in 1814 (Bast, 1928; Parent, 2012), in 1842 Burdach published *Comparative Psychologie* (Comparative Psychology) in two volumes, which represented the first two books of his *Blicke ins Leben* (Views on Life) in four volumes, published between 1842 and 1848 (Burdach, 1842a,b).

^6^Known in Italian as Istituto Veneto di Scienze, Lettere e Arti, the institute was founded in 1838 and is still active today (https://www.istitutoveneto.it).

^7^Known in Italian as Regia Accademia Scientifico-Letteraria di Milano, this university was founded in 1859 and merged in 1924 with the Regia Università degli Studi di Milano (Royal University of Milan).

^8^This is Volume III of Prichard’s work. In the previous Volume I, Prichard stated: "The comparative physiology and psychology of men had never been made expressly the subject of inquiry, until the publication of my work" (Prichard, 1836). Nevertheless, he never employed the exact phrase comparative psychology in that volume.

^9^In 1884 Giuseppe Sergi, Professor of Anthropology at University of Bologna from 1880, moved to University of Rome, where he became Professor of Anthropology under the Faculty of Science (Cimino and Foschi, 2012). In 1885, in the palace of the Royal School of Application for Engineers, Sergi founded a laboratory of anthropology, a section of which performed psychological research (Misiak and Staudt, 1954). When, in 1886, the British biologist and psychologist Francis Dalton (1822–1911), cousin of Charles Darwin, visited Sergi’s laboratory, he was highly impressed by the quantity and quality of Sergi’s instruments, as the Hipp chronoscope for the measurement of reaction times to external stimuli (Cerro, 2017). In 1887 Sergi founded, in the Collegio Romano palace (which was an important Gesuit school from 1584 to 1870 and currently hosts the headquarter of the Italian Ministry of Culture), the Institute of Anthropology and the Museum of Anthropology (Soncin et al., 2017). In 1889, the psychological laboratory became autonomous and acquired the name Laboratory of Experimental Psychology (Cicciola and Foschi, 2017). In the same year, the institute changed name into Institute of Anthropology and Experimental Psychology (Cicciola and Foschi, 2017). Due to the international recognition of his work, in 1905 Sergi became President of the Fifth International Congress of Psychology, which was held in Rome (Cerro, 2017).
